# Enhanced Fungicidal Efficacy by Co-Delivery of Azoxystrobin and Diniconazole with Cauliflower-Like Metal–Organic Frameworks NH_2_-Al-MIL-101

**DOI:** 10.3390/ijms221910412

**Published:** 2021-09-27

**Authors:** Huiping Chen, Yongpan Shan, Lidong Cao, Pengyue Zhao, Chong Cao, Fengmin Li, Qiliang Huang

**Affiliations:** 1 Key Laboratory of Integrated Pest Management in Crops, Ministry of Agriculture, Institute of Plant Protection, Chinese Academy of Agricultural Sciences, No. 2 Yuanmingyuan West Road, Haidian District, Beijing 100193, China; hpingchen@126.com (H.C.); shanyongpan@yeah.net (Y.S.); pengyue_8825@163.com (P.Z.); ccao@ippcaas.cn (C.C.); fmli@ippcaas.cn (F.L.); 2 State Key Laboratory of Cotton Biology, Institute of Cotton Research, Chinese Academy of Agricultural Sciences, No. 38 Yellow River Avenue, Anyang 455000, China

**Keywords:** NH_2_-Al-MIL-101, controlled release, dual encapsulation, synergistic effect, rice sheath blight

## Abstract

Long-term use of a single fungicide increases the resistance risk and causes adverse effects on natural ecosystems. Controlled release formulations of dual fungicides with different modes of action can afford a new dimension for addressing the current issues. Based on adjustable aperture and superhigh surface area, metal–organic frameworks (MOFs) are ideal candidates as pesticide release carriers. This study used Al^3+^ as the metal node and 2-aminoterephthalic acid as the organic chain to prepare aluminum-based metal–organic framework material (NH_2_-Al-MIL-101) with “cauliflower-like” structure and high surface area of 2359.0 m^2^/g. Fungicides of azoxystrobin (AZOX) and diniconazole (Dini) were simultaneously encapsulated into NH_2_-Al-MIL-101 with the loading content of 6.71% and 29.72%, respectively. Dual fungicide delivery system of AZOX@Dini@NH_2_-Al-MIL-101 demonstrated sustained and pH responsive release profiles. When the maximum cumulative release rate of AZOX and Dini both reached about 90%, the release time was 46 and 136 h, respectively. Furthermore, EC_50_ values as well as the percentage of inhibition revealed that AZOX@Dini@NH_2_-Al-MIL-101 had enhanced germicidal efficacy against rice sheath blight (*Rhizoctonia solani*), evidenced by the synergistic ratio of 1.83. The present study demonstrates a potential application prospect in sustainable plant protection through co-delivery fungicides with MOFs as a platform.

## 1. Introduction

Rice is the main food in many countries, whose production is easily affected by biological pathogens [1,2]. Rice sheath blight, caused by *Rhizoctonia solani* Kühn AG1-1A (*R. solani*), is one of the most damaging diseases of rice, and is only second in importance to rice blast [3,4]. It occurs in temperate and tropical production areas, and is most prominent in areas where rice is grown under conditions including planting semi-dwarf cultivars, high planting density, and high nitrogen fertilization [5,6]. It is estimated that the yield loss of rice ranging from 4% to 50% can be attributed to the sheath blight [7,8,9]. Fungicides are the most widely used method to control sheath blight, which act on *R. solani* and its sclerotia in various ways. A review introduced some commercially available fungicides to control rice sheath blight such as azoxystrobin, carbendazim, hexaconazole, etc. [2]. Efficacy of several fungicide combinations have been summarized by Chaudhary et al. [10] as well as Uppala and Zhou [11] such as tricyclazole and propiconazole, azoxystrobin, difenoconazole, fluxapyroxad, epixiconazole, etc. In the process of controlling rice sheath blight, the main formulations are aqueous suspension concentrate, emulsifiable concentrate, water dispersible granule, wettable powder, and the different application methods are foliar spraying, seed treatment, and soil application [12,13]. Furthermore, succinate dehydrogenase inhibitors, quinone outside inhibitors, and ergosterol biosynthesis inhibitors are the three main classes of fungicides [9,14]. Strobilurin fungicides have been the backbone for combating rice sheath blight. Strobilurin fungicide of azoxystrobin (AZOX) is widely used because it works effectively against pathogen infestation [15,16]. AZOX, which belongs to a quinone outside inhibitor, arrests fungal growth via disrupting the electron transport chain, preventing adenosine triphosphate synthesis, and restricting respiration in fungi [17]. In addition, diniconazole (Dini) is reported to be effective in reducing sheath blight and increasing grain yield [18]. It is a systemic fungicide that is toxic to a broad range of fungal species, and inhibits the biosynthesis of ergosterol in fungal species by blocking the 1,4-α-demethylase enzyme [19,20].

However, long-term use of a single fungicide increases the risk of resistance to fungicides. On the other hand, excessive use of fungicides can harm human health and natural ecosystems [2]. Accordingly, mixtures of fungicides are applied to crops as a resistance management strategy. In addition, controlled release formulations (CRFs) of fungicides can afford a new dimension for addressing the current issues. CRFs aim to decrease agrochemical input, enhance pesticide utilization efficiency, improve stability, and reduce adverse effects on the environment [21,22].

The performance of CRFs on controlling the release of fungicide is closely related to the carrier materials. As an emerging class of porous materials, metal–organic frameworks (MOFs) with unique properties have been applied in many fields. At present, studies have been conducted on the use of MOFs in pesticides such as pesticide sensing [23], delivery [24,25,26], adsorption, and photodegradation [27]. In our previous work, we also prepared pesticide-loaded Fe-MIL-100 [28] and NH_2_-Fe-MIL-101 [29] and studied the controlled release profiles of AZOX and Dini, respectively. However, the use of MOFs in loading of dual pesticides has not been reported. Dual encapsulation has been proven to have a synergistic effect of dual-loaded fungicides [30].

Apart from adjustable aperture, superhigh surface area and remarkable functionality, luminescence of MOFs is also a commonly reported property [31]. Recently, various modified MOFs with luminescence have been synthesized by introducing functional groups into organic ligands or combining lanthanide ions [32,33]. Among the plethora of MOFs, aluminum-containing MOFs (Al–MOFs) have auto-fluorescence characteristics [34], which helps to use the fluorescence characteristics of the carrier material to observe the transmission and distribution of the drug-loaded system in the target. Moreover, aluminum salts are inexpensive and readily available [35]. Thus, Al–MOFs have great prospect as carriers in the smart delivery of pesticides, and the use of Al–MOFs in loading of dual pesticides is also promising.

In this work, with Al^3+^ as the metal node and 2-aminoterephthalic acid as the organic chain, a microwave-assisted solvothermal method was used to prepare fluorescent NH_2_-Al-MIL-101 with a cauliflower-like structure, and AZOX and Dini were simultaneously loaded. In addition, the controlled and sustained release behaviors of AZOX and Dini from pesticide-loaded NH_2_-Al-MIL-101 as well as the fungicidal bioactivities against rice sheath blight were investigated. This study aimed to obtain fungicide-loaded granules by loading two active ingredients with different mechanisms of action into cauliflower-like NH_2_-Al-MIL-101, hoping that the fungicide-loaded granules have a slow-release capability and high antifungal activity against *R. solani*. Moreover, this study also tries to provide a novel nanocarrier platform for the potential application of pesticides in sustainable plant protection.

## 2. Results and Discussion

### 2.1. Sample Preparation and Characterization

**Figure 1:**
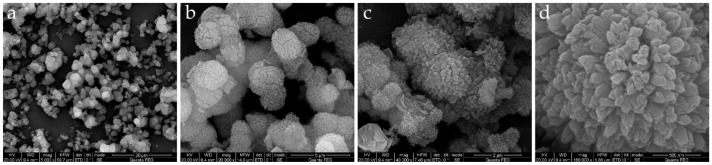
The scanning electron microscope (SEM) images of the NH2-Al-MIL-101 at magnification 5000× (**a**), 20,000× (**b**), 40,000× (**c**) and 160,000× (**d**).

**Figure 2:**
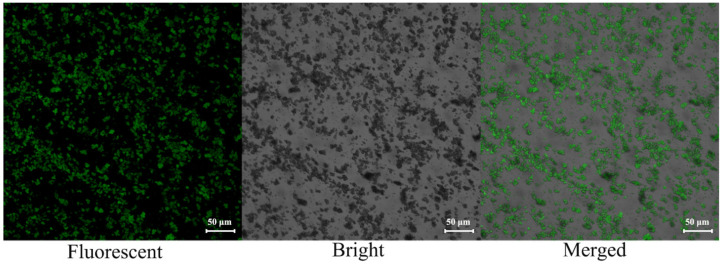
The laser confocal microscope (CLSM) images of NH_2_-Al-MIL-101.

NH_2_-Al-MIL-101 was synthesized by a microwave irradiation method using Al^3+^ as the node and 2-aminoterephthalic acid as the organic ligand. The scanning electron microscope (SEM) images of NH_2_-Al-MIL-101 shown in Figure 1 indicated the morphology of crystals with a cauliflower structure, and there were many pores on the surface, facilitating the loading of pesticide molecules. At the same time, the fluorescence characteristics of the prepared carrier materials were observed with a confocal laser microscope (CLSM). Figure 2 shows that the material exhibited a strong fluorescence signal when excited at 488 nm. In addition, the average particle sizes of NH_2_-Al-MIL-101 and AZOX@Dini@NH_2_-Al-MIL-101 were 1.88 ± 0.10 and 1.56 ± 0.11 μm, respectively, with both of the PDI values less than 0.5. These particle sizes were measured using a ZetaSizer Nano ZS Analyzer based on dynamic light scattering.

**Figure 3:**
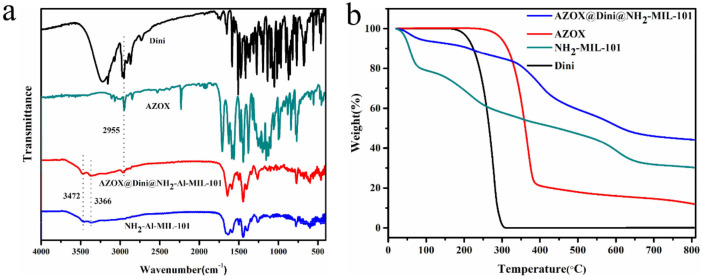
Fourier transform infrared (FTIR) spectra (**a**) and thermogravimetric analyses (TGA) (**b**) of azoxystrobin (AZOX) and diniconazole (Dini) standards, NH_2_-Al-MIL-101, and AZOX@Dini@NH_2_-Al-MIL-101.

The Fourier transform infrared (FT-IR) spectra of AZOX and Dini standards, NH_2_-Al-MIL-101 and AZOX@Dini@NH_2_-Al-MIL-101 are shown in Figure 3a. The peak of NH_2_-Al-MIL-101 at 1327 cm^−1^ corresponded to the C–N stretching vibration on the aromatic ring. The absorption peak at 3472 and 3366 cm^−1^ can be attributed to the asymmetrical and symmetrical stretching vibration of the amine groups in NH_2_-Al-MIL-101, indicating that NH_2_-Al-MIL-101 with 2-aminoterephthalic acid as the organic chain was successfully prepared [36]. After loading AZOX and Dini, a peak at 2955 cm^−1^ of AZOX@Dini@NH_2_-Al-MIL-101, corresponding to the C–H stretching vibration, could be observed in AZOX and Dini, indicating the successful loading of AZOX and Dini into NH_2_-Al-MIL-101.

Thermogravimetric analyses (TGA) are always used to explore the thermal stability and decomposition behaviors of materials. The TGA curves of the AZOX and Dini standards, NH_2_-Al-MIL-101, and AZOX@Dini@NH_2_-Al-MIL-101 are depicted in Figure 3b. The degradation of NH_2_-Al-MIL-101 can be roughly divided into three parts. Water was the first step, followed by some of the DMF, which is consistent with the literature [37]. Finally, it might be the result of the decomposition of Al_2_O_3_ [38]. More weight was lost by AZOX@Dini@NH_2_-Al-MIL-101 than NH_2_-Al-MIL-101 between 150 and 600 °C, indicating the successful loading of AZOX and Dini.

**Figure 4:**
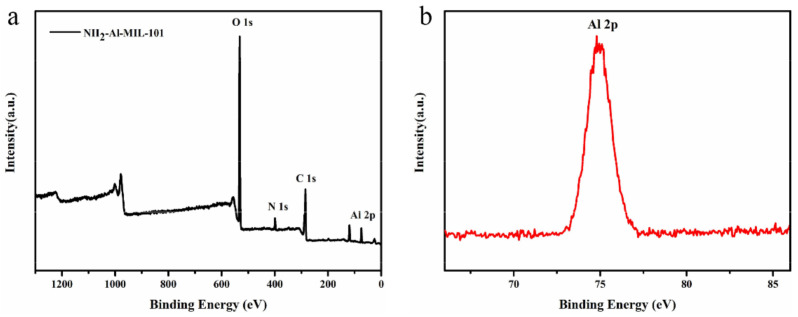
X-ray photoelectron spectroscopy (XPS) spectra of NH_2_-Al-MIL-101 (**a**) and Al 2p (**b**).

The elemental information on the surface of the materials could be obtained from the X-ray photoelectron spectroscopy (XPS) spectra. The XPS spectra of NH_2_-Al-MIL-101 are presented in Figure 4. In the spectrum of NH_2_-Al-MIL-101, the binding energies of approximately 284.8, 399.4, 532.3, and 74.9 eV belonged to C 1s, N 1s, O 1s, and Al 2p, respectively, which showed that the carrier contained carbon, nitrogen, oxygen, and aluminum elements.

**Figure 5:**
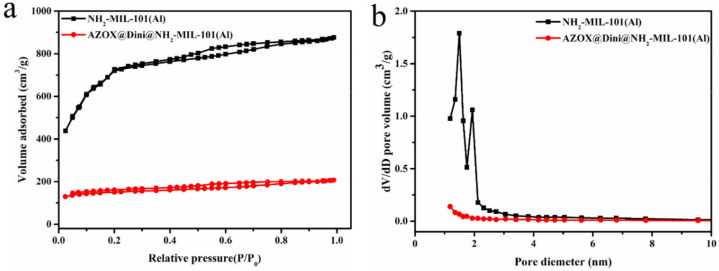
Nitrogen adsorption−desorption isotherms (**a**) and pore size distributions (**b**) of NH_2_-Al-MIL-101 and AZOX@Dini@NH_2_-Al-MIL-101.

Figure 5 and Table 1 showed the N_2_ adsorption–desorption isotherms and pore size distributions of NH_2_-Al-MIL-101 and AZOX@Dini@NH_2_-Al-MIL-101. As the pressure increases, the cavities of the MOF became filled [37]. However, the isotherm of NH_2_-Al-MIL-101 was slightly different to the well-known characteristic steps of the typical structure of MIL-101. In addition, the material exhibited a typical H4 hysteresis loop, which was characteristic for material containing both micro- and mesopores [39,40].

**Figure 6:**
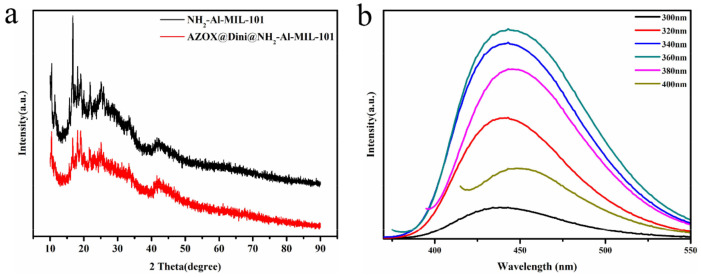
(**a**) Powder X-ray diffraction (XRD) patterns of NH_2_-Al-MIL-101 and AZOX@Dini@NH_2_-Al-MIL-101; (**b**) Emission spectra of NH_2_-Al-MIL-101 under different excitation wavelengths.

According to N_2_ adsorption/desorption analysis, NH_2_-Al-MIL-101 had a high surface area and a large pore volume. After loading AZOX and Dini, the pores of NH_2_-Al-MIL-101 were filled, and the S_BET_ and V_t_ were reduced from 2359.0 to 468.7 m^2^/g and from 1.36 to 0.32 cm^3^/g, respectively. This phenomenon indicated that during the process of loading pesticides on NH_2_-Al-MIL-101, pesticides occupied its mesoporous structure, resulting in a decrease in specific surface area and pore size. Strangely, the BET surface areas of NH_2_-Al-MIL-101 were slightly different from those previously reported of 1968 or 2073.8 m^2^/g, which is probably due to the replacement of AlCl_3_•6H_2_O with AlCl_3_ (anhydrous salt) [38,41]. Furthermore, powder X-ray diffraction (XRD) confirmed that the crystal structure of NH_2_-Al-MIL-101 remained basically unchanged after loading AZOX and Dini (Figure 6a).

In order to determine the autofluorescence characteristics of the prepared materials, the emission spectra of the NH_2_-Al-MIL-101 aqueous suspension under different excitation wavelengths were measured using a Fluoromax-4 fluorescence spectrometer. As shown in Figure 6b, there were emission spectra with different intensities under different excitation wavelengths of 300, 320, 340, 360, 380, and 400 nm, and the emission spectrum was the strongest at the excitation wavelength of 360 nm. Autofluorescent MOF has a label-free nature, combining the inherent photophysical properties and porosity to encapsulate objects, which avoids the introduction of the other toxic side effects of fluorescent modification [42].

### 2.2. Optimization of the Loading Content and Encapsulation Efficiency

As listed in Table 2, AZOX and Dini were loaded separately or simultaneously under the pesticide–carrier mass ratio of 1:1. When AZOX and Dini were loaded separately, the loading contents were 7.28% and 29.79%, respectively; when AZOX and Dini were loaded simultaneously, the loadings were 6.71% and 29.72%, respectively. The results showed that there was no significant difference in the loading content of both AZOX and Dini into the synthesized NH_2_-Al-MIL-101 when compared with loading AZOX and Dini separately. In comparison to our earlier study on single loaded systems of AZOX [28] and Dini [29] into the Fe-MIL-100 and NH_2_-Fe-MIL-101, the results revealed that the amount of pesticide loaded did not relate to the metal ions, but mainly depended on the size of the pore, which was consistent with the loading of ibuprofen into MIL-53 (Cr, Fe) with different metal ions [43]. Therefore, we prepared a double pesticide-carrying system (AZOX@Dini@NH_2_-Al-MIL-101) for sample characterization and bioactivity determination.

### 2.3. Controlled Release of AZOX and Dini

**Figure 7:**
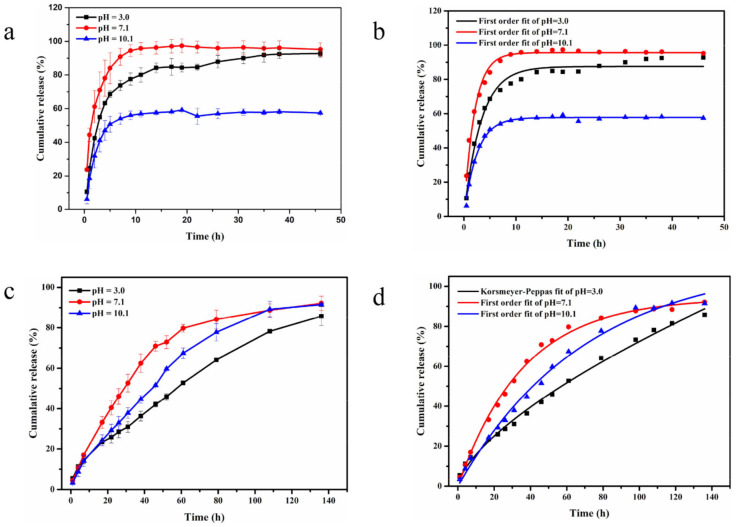
The curves of AZOX release from AZOX@Dini@NH_2_-Al-MIL-101 (**a**) and fitting plots using first-order equation at different pH values of 3.0, 7.1, and 10.1 (**b**); the curves of Dini release from AZOX@Dini@NH_2_-Al-MIL-101 (**c**) and fitting plots using first-order equation at different pH values of 7.1, 10.1, and Korsmeyer–Peppas model at 10.1 (**d**). Error bars correspond to standard errors of triplicate measurements.

The fungicides of AZOX and Dini are usually used to control plant diseases, and most plant juices are weakly acidic or alkaline [44]. Therefore, release media with three different pH values of 3.0, 7.1, and 10.1 were selected to explore the pH sensitive release profiles of AZOX and Dini from AZOX@Dini@NH_2_-Al-MIL-101. As shown in Figure 7a, the release of AZOX from AZOX@Dini@NH_2_-Al-MIL-101 showed a slower release rate under alkaline conditions. After 46 h, the cumulative release was greater than 90% under acidic (pH 3.0) and neutral (pH 7.1) conditions, however, only about 50% was released under alkaline conditions of pH 10.1. As shown in Figure 7c, compared with the neutral and alkaline conditions, the release of Dini from AZOX@Dini@NH_2_-Al-MIL-101 showed a slower release rate under acidic conditions. After 136 h, the release of Dini under different pH conditions reached the peak, and the cumulative release was about 86% (pH 3.0), 92% (pH 7.1), and 91% (pH 10.1), respectively. From the single factor of AZOX or Dini, this was consistent with previous reports, where the higher the solubility of the active molecule was at a certain pH, the greater the release rate [45]. From an overall perspective, by comparing the time for the cumulative release of AZOX and Dini to reach the maximum, it was found to be closely related to the drug loading. The difference of release behaviors between AZOX and Dini was probably attributed to the solubility of cargo molecules in release media. However, according to the cumulative curve analysis of AZOX and Dini, their release can be roughly divided into two stages of initial burst release and followed sustained release.

By fitting the release data of the AZOX and Dini into the four commonly used mathematical models, the release kinetics were studied. The zero-order model (Equation (1)), first-order model (Equation (2)), Higuchi model (Equation (3)), and Korsmeyer–Peppas model (Equation (4)) are shown below, where M_t_/M_∞_ is the fractional active agent released at time t, k is a constant, and *n* is related to the release mechanism [46]. For the Korsmeyer–Peppas model, when *n* ≤ 0.45, the release follows Fickian diffusion (case diffusional); when 0.45 < *n* ≤ 0.89, it means anomalous (non-Fickian) transport, and when *n* > 0.89, the release follows a super Case II transport [47,48]. The linear fitting results of AZOX and Dini are presented in Figure 7b,d, respectively. The calculated correlation coefficient (R^2^) (Table 3) of the release data revealed that the release kinetics of AZOX@Dini@NH_2_-Al-MIL-101 fitted well to the first-order compared to the other models, except Dini from AZOX@Dini@NH_2_-Al-MIL-101 at pH 3.0. In addition to the first-order kinetic equation, the Korsmeyer–Peppas model can also fit well in most cases.

(1) $$\frac{M_{t}}{M_{\infty }}=kt$$

(2) $$\ln(1-\frac{M_{t}}{M_{\infty }})=-kt$$

(3) $$\frac{M_{t}}{M_{\infty }}=k\sqrt{t}$$

(4) $$\ln\frac{M_{t}}{M_{\infty }}=n\ln t+\ln k$$

To study the release mechanism of AZOX and Dini from AZOX@Dini@NH_2_-Al-MIL-101, more information could be obtained from the release exponent (*n*) of the Korsmeyer–Peppas model. For AZOX from AZOX@Dini@NH_2_-Al-MIL-101, *n* values were 0.25, 0.17, and 0.20 at pH 3.0, 7.1, and 10.1, respectively, indicating that the release of AZOX from AZOX@Dini@NH_2_-Al-MIL-101 conformed to Fickian diffusion. That is, the release behavior could be described by the first-order model. For Dini from AZOX@Dini@NH_2_-Al-MIL-101, *n* was 0.66, 0.46, and 0.63, respectively. Under acidic conditions, the release of Dini from the carrier followed the Korsmeyer–Peppas model. When 0.45 < *n* ≤ 0.89, it is anomalous (non-Fickian) transport, which shows the coexistence of diffusion and erosion. Under neutral and alkaline conditions, the first-order kinetics model was considered relevant. Hence, it shows that the overall reactions are dependent upon the concentration of Dini. According to the *n*, these were 0.46 (pH 7.1) and 0.63 (pH 10.1), respectively. Although the best fits have been with the first-order model, the process is probably much more complex.

### 2.4. In Vitro Antifungal Activity Assay

**Figure 8:**
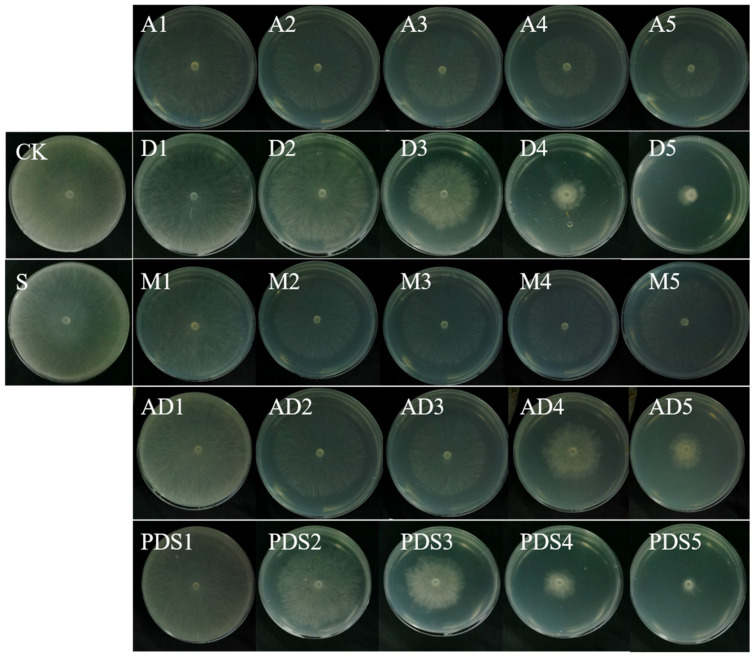
Images of the fungicidal activities of the control without solvent (CK), solvent (S), pure AZOX (A1~5, 0.007, 0.033, 0.17, 0.83, and 4.14 mg/L), pure Dini (D1~5, 0.0033, 0.017, 0.083, 0.42, and 2.08 mg/L), blank carrier of NH_2_-Al-MIL-101 (M1~5, 31.7, 63.4, 26.7, 253.4, and 506.8 mg/L), AZOX + Dini (AD1~5, 0.005, 0.02, 0.1, 0.5, and 2.5 mg/L), AZOX@Dini@NH_2_-Al-MIL-101 (PDS1~5, 0.003, 0.016, 0.08, 0.41, and 2.07 mg/L) against rice sheath blight (*R. solani*).

**Figure 9:**
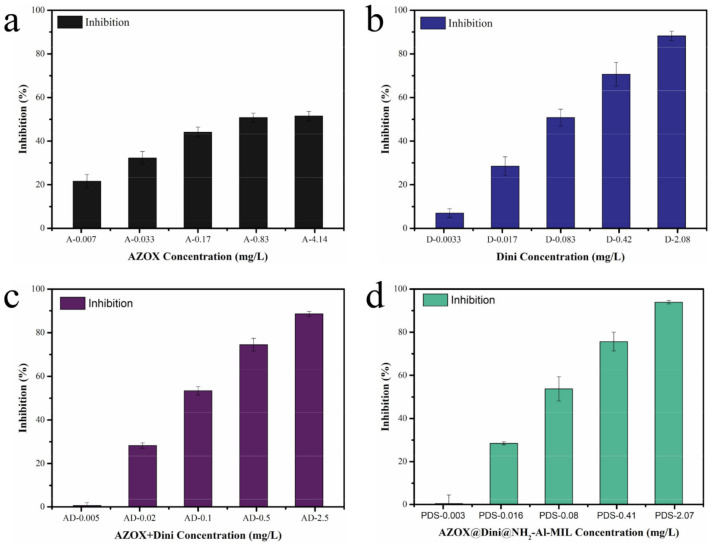
Inhibition of rice sheath blight in pure AZOX (**a**), pure Dini (**b**), AZOX + Dini (**c**), and AZOX@Dini@NH_2_-Al-MIL-101 (**d**) at 25 ± 2 °C. The error bars represent standard deviation of the mean.

The in vitro antifungal studies were performed with several treatments: a control, where the mycelia were plated on the PDA with only solvent (S), without solvent (CK), the NH_2_-Al-MIL-101 (M), pure AZOX (A), pure Dini (D), a physical mixture of AZOX and Dini in the same ratio as the active ingredients in the pesticide-loaded system (AZOX:Dini = 1:4.6, AZOX + Dini, AD), and AZOX@Dini@NH_2_-Al-MIL-101(PDS). Except for S and CK, each treatment was performed with five gradient concentrations, and each concentration was repeated five times. The images of colonies are shown in Figure 8. The antifungal activity was analyzed using the mycelia growth method. The inhibitory effect was evaluated based on the inhibition rate (Figure 9) and the calculated EC_50_ (Table 4).

As is well-known, the lower the EC_50_ value, the more effective the fungicide in combating rice sheath blight (*R. solani*). The EC_50_ of the fungicides is presented in Table 4. The results showed that pure Dini had a higher antifungal activity against *R. solani* compared to pure AZOX. This result could be attributed to the fact that AZOX has been used in large quantities for a long time and has developed resistance to *R. solani*. Moreover, the EC_50_ value of pure AZOX and Dini were 1.251 and 0.099, respectively. Frequent use of a single fungicide might easily lead to the development of the resistance of crop pathogens to fungicides. A common resistance management strategy is to apply the mixtures of fungicides with different modes of action [49]. It has been reported that AZOX (18.2%) combined with difenoconazole (11.4%) had a lower risk of pesticide resistance in pathogens [50]. Interestingly, the physical mixture and the synthesized AZOX@Dini@NH_2_-Al-MIL-101 showed a remarkably better antifungal activity on rice sheath blight with lower EC_50_ values. Furthermore, the EC_50_ value of AZOX@Dini@NH_2_-Al-MIL-101 was lower than AD. In general, SR > 1.5 indicates that the two agents have a synergistic effect, and the SR value between 0.5 and 1.5 indicates that the two agents are additive. According to Table 4, the SR value of AD was additive, and the antifungal effect of AZOX@Dini@NH_2_-Al-MIL-101 showed that it was synergistic. Interestingly, the blank carrier NH_2_-Al-MIL-101 also showed certain fungicidal activity against *R. solani*. In the control experiment, NH_2_-Al-MIL-101 also showed a certain antibacterial activity, whose maximum inhibition rate from 31.68 ppm to 506.8 ppm was 14.38%. The previous literature studies also reported that the complexation of the ligand to the aluminum center has antibacterial/antifungal activity [51].

The synergistic interaction is the combined effect of multiple factors rather than a single specific effect [52]. It has been reported that the combination of AZOX and other active ingredients (such as metominostrobin, difenoconazole) had a good antifungal effect [17,53]. Similarly, the EC_50_ of AD was less than AZOX or Dini, but greater than AZOX@Dini@NH_2_-Al-MIL-101, implying that the NH_2_-Al-MIL-101 also contributed to the antifungal effect.

## 3. Materials and Methods

### 3.1. Materials

Aluminum chloride (AlCl_3_, 98%) and N,N-dimethylformamide (DMF) were purchased from Beijing Ouhe Technology Co. Ltd. (Beijing, China). 2-Aminoterephthalic acid (H_2_ATA) was obtained from Aladdin Reagent Co. Ltd. (Shanghai, China). The Pesticide Bioassay Lab in the Institute of Plant Protection of the Chinese Academy of Agricultural Sciences generously provided the rice sheath blight fungus (*R. solani*). All other chemicals were commercially available and used without additional purification.

### 3.2. Synthesis of the Particles

#### 3.2.1. Synthesis of NH_2_-Al-MIL-101 Crystals

The synthesis of NH_2_-Al-MIL-101 crystals used a microwave radiation method, which was slightly different from the previously reported steps [54,55]. Briefly, approximately 0.51 g of AlCl_3_ and 0.56 g of H_2_ATA were dispersed in 30 mL of DMF ultrasonically. Then, the well-mixed mixture was transferred into a Teflon-lined stainless autoclave, sealed, and placed in a microwave oven (XH-800G, Beijing Xianghu Science and Technology Development Co. Ltd., Beijing, China). The autoclave was heated at 130 °C and 400 W for 4 h by microwave irradiation. After the reaction was done, NH_2_-Al-MIL-101 was collected by centrifugation (10,000 r/min, 10 min), followed by washing with fresh acetone three times. The sample was activated in boiling methanol for 12 h to remove the organic matter in the pores. Finally, NH_2_-Al-MIL-101 were dried overnight under vacuum at 80 °C for further characterization and analysis.

#### 3.2.2. Preparation of AZOX@Dini@NH_2_-Al-MIL-101 Crystals

AZOX and Dini were simultaneously loaded into NH_2_-Al-MIL-101 nanocrystals by physical adsorption. First, each 30 mg of Dini, AZOX, and NH_2_-Al-MIL-101 (1:1:1) were added to the 10 mL of centrifuge tube separately, and 1 mL of dichloromethane was added. Subsequently, the plastic centrifuge tube was sealed and stirred at room temperature in the dark for 6 h. The mixture was centrifuged at 10,000 r/min for 10 min and the supernatant was discarded. Finally, the dual fungicide-loaded MOFs (AZOX@Dini@NH_2_-Al-MIL-101) were dried at 50 °C before further characterization and application.

The loading content and encapsulation efficiency of AZOX and Dini from AZOX@Dini@NH_2_-Al-MIL-101 were measured by the ultrasonic dissolution method. Approximately 10 mg of the prepared AZOX@Dini@NH_2_-Al-MIL-101 nanocrystals were weighed into 25 mL of methanol and extracted by ultrasonication for 3 h. Then, the concentration of the supernatant was measured by high-performance liquid chromatography (HPLC, 1200-DAD (Diode Array Detector), Agilent, Santa Clara, CA, USA). The HPLC operating conditions were as follows: Extend-C_18_ reversed-phase column (5 µm, 4.6 × 250 mm) with methanol/0.1% formic acid aqueous solution as the mobile phase with gradient elution: 0~6 min (*v*/*v*, 70:30), 7~14 min (*v*/*v*, 85:15), 15~16 min (*v*/*v*, 70:30); column temperature, 27 °C; flow rate, 1.0 mL/min; injection volume, 5 µL; and detection wavelength, 254 nm. The loading content and encapsulation efficiency of AZOX and Dini were calculated by the formulas reported by us [28].

### 3.3. Characterizations of Samples

To identify the chemical structures of the samples, the FTIR spectra of AZOX, Dini, NH_2_-Al-MIL-101, and AZOX@Dini@NH_2_-Al-MIL-101 were conducted on a spectrometer with a potassium bromide pellet, in the range 400–4000 cm^−1^ (NICOLET 6700, Thermo Scientific, Waltham, MA, USA). The morphological feature was characterized with a scanning electron microscope (SEM, SU8000, Hitachi, Ltd., Tokyo, Japan) at an accelerating voltage of 30 kV. Meanwhile, the fluorescence characteristic of the prepared carrier material was also observed under a laser confocal microscope (CLSM, Zeiss LSM 880, Carl Zeiss AG, Germany).

To confirm the thermal stability of the samples, TGA was carried out using a PerkinElmer Pyris Diamond (Woodland, CA, USA) from 20 to 550 °C with the heating rate of 20 °C/min under a N_2_ atmosphere. The elemental compositions of the samples were analyzed by X-ray photoelectron spectroscopy (XPS, ESCALab 250Xi, Thermo Fisher Scientific, Waltham, MA, USA). To study the crystal structure of the sample, powder X-ray diffraction analysis (XRD) was performed on a Bruker D8 Advance X-ray diffractometer (Bruker, Karlsruhe, Germany) with Cu Kα radiation (λ = 0.15418 nm).

The nitrogen adsorption/desorption isotherms and pore structure of the samples were studied with a specific surface area and pore size analyzer (TriStarII 3020, Micromeritics Instruments Corp, Norcross, GA, USA). The sample was degassed at 120 °C for 6 h, and the saturation pressure was measured with nitrogen as the analysis gas. The specific surface area of the sample was determined by the Brunauer–Emmett–Teller (BET) equation, and the pore size and pore size distribution were calculated by the Barrett–Joyner–Halenda (BJH) model.

The fluorescence intensity of NH_2_-Al-MIL-101 aqueous solution was measured by a fluorescence spectrometer (FluoroMaxR-4, HORIBA, Jobin Yvon, France).

### 3.4. In Vitro Release of AZOX and Dini

The controlled release characteristics of AZOX and Dini from the prepared AZOX@Dini@NH_2_-Al-MIL-101 were studied under three release media with different pH values (3.0, 7.1, and 10.1) containing phosphate buffered saline (PBS), ethanol, and Tween-80 emulsifier (70:29.5:0.5, *v*/*v*/*v*). Briefly, approximately 40 mg of AZOX@Dini@NH_2_-Al-MIL-101 was dispersed in 2.0 mL of release medium in dialysis bags (molecular weight cut-off: 8000–14,000 Da). The sealed dialysis bag was thereafter placed in 200 mL of release medium at 30 ± 2 °C and stirred at 150 r/min using a thermostatic oscillator (HZQX300C, Shanghai Yiheng Scientific Instrument Co. Ltd., Shanghai, China). For HPLC analysis, 0.8 mL of the release medium was withdrawn at the given time intervals, and the same volume of fresh buffer solution was supplied to ensure the constant volume of release medium. All the treatment was repeated three times. The accumulative release was calculated as Equation (5).

(5) $$Er=\frac{V_{e}\sum_{i=0}^{n-1}C_{i}+V_{0}C_{n}}{m_{\mathrm{pesticide}}}\times 100\%$$

where *Er* was the accumulative release (%) of AZOX/Dini from AZOX@Dini@NH_2_-Al-MIL-101; *V_e_* was the volume of the release medium taken at a given time interval (*V_e_* = 0.8 mL); *V_0_* was the volume of release solution (200 mL); *C_n_* (mg/mL) was the AZOX/Dini concentration in the release medium at time *n*; and *m_pesticide_* (mg) was the total pesticide loaded in the particles. The measurements were performed in triplicate.

### 3.5. Bioactivity Studies of the Samples

The bioactivity of the synthesized AZOX@Dini@NH_2_-Al-MIL-101 was evaluated against rice sheath blight using a poisoned medium technique with potato dextrose agar (PDA) as the medium. The PDA was amended in five different conditions (pure AZOX, pure Dini, NH_2_-Al-MIL-101, a physical mixture of AZOX and Dini as well as the synthesized particles) at five gradient concentrations, which were prepared in dimethyl sulfoxide (DMSO). Meanwhile, mycelia plated on the PDA with the solvent of DMSO and without the solvent both served as controls. The 5 mm mycelial disc from the margins of the actively growing culture of rice sheath blight was placed at the center of PDA with and without treatments. Each treatment was repeated five times. After two days of incubation at 25 ± 2 °C, the percentage inhibition (%) of the radial growth by the fungicidal was calculated as follows: percentage inhibition (%) = (colony diameter of control – colony diameter of treatment)/(colony diameter of control – l5 mm) × 100%.

The probability value analysis method was used to calculate the effective inhibitory concentration (*EC*_50_) of each treatment on the growth of the target fungus hyphae, and the synergistic ratio (*SR*) of the mixture was calculated by the following formula [51] as Equations (6) and (7):

(6) $${\mathrm{EC}}_{50}(th)=\frac{a+b}{\frac{a}{{\mathrm{EC}}_{50}(AZOX)}+\frac{b}{{\mathrm{EC}}_{50}(Dini)}}$$

(7) $$SR=\frac{{\mathrm{EC}}_{50}(th)}{{\mathrm{EC}}_{50}(ob)}$$

where *a* represents the proportion of *AZOX* in the compound drug; *b* represents the proportion of *Dini* drug in the compound drug; *EC*_50_*(th)* is the theoretical inhibitory concentration of the compound drug against pathogens; and *EC*_50_*(ob)* is the observed inhibitory concentration of the compound drug against pathogens. The *SR* value of each set ratio formula is calculated by two formulas. *SR* > 1.5 indicates that the two agents have a synergistic effect, and the *SR* value between 0.5 and 1.5 indicates that the two agents are additive, and its value is less than 0.5 for antagonism.

### 3.6. Statistical Analysis

Data are presented as mean ± standard deviation (SD) and the statistical difference of the parameters was analyzed using one-way analysis of variance (ANOVA) and Duncan’s multiple range tests (*p* ≤ 0.05) by SPSS software.

## 4. Conclusions

In summary, this study used Al^3+^ as the metal node and 2-aminoterephthalic acid as the organic chain to prepare aluminum-based metal–organic framework material (NH_2_-Al-MIL-101) with a “cauliflower-like” structure. The loading of AZOX and Dini into NH_2_-Al-MIL-101 was through physical adsorption due to the large specific surface area and pore volume. The prepared samples were characterized by SEM, XPS, FTIR, TGA, and N_2_ adsorption/desorption isotherms; the autofluorescence characteristics of NH_2_-Al-MIL-101 were studied by fluorescence spectroscopy and CLSM. Moreover, AZOX@Dini@NH_2_-Al-MIL-101 showed a pH sensitive and sustained release, when the maximum cumulative release rate of AZOX and Dini both reached about 90%, and the release time was 46 and 136 h, respectively. Meanwhile, the release kinetics of AZOX@Dini@NH_2_-Al-MIL-101 fitted well to the first-order compared to the other models used in this work, except Dini from AZOX@Dini@NH_2_-Al-MIL-101 at pH 3.0. Furthermore, EC_50_ values as well as the percentage of inhibition revealed that AZOX@Dini@NH_2_-Al-MIL-101 had enhanced germicidal efficacy against rice sheath blight as evidenced by the synergistic ratio of 1.83. The present NH_2_-Al-MIL-101 carrier is relatively modest in ameliorating the defect of pesticides and is still a proof of concept. Therefore, optimizing its preparation method and particle size is also the key to preventing crop diseases [56]. As the research and development of new pesticides is increasingly difficult, the demand for pesticides in production is becoming more and more diverse, so the status of pesticide mixtures that meet the needs of production has become more and more important. The present study demonstrates a potential application prospect in sustainable plant protection through co-delivery fungicides with a different mode of action.

## Figures and Tables

**Table 1:** Characterization of mesoporous structure of NH_2_-Al-MIL-101 and AZOX@Dini@NH_2_-Al-MIL-101.

Samples	S_BET_ (m^2^/g)	V_t_ (cm^3^/g)	D_BJH_ (nm)
NH_2_-Al-MIL-101	2359.0	1.36	2.30
AZOX@Dini@NH_2_-Al-MIL-101	468.7	0.32	2.73

S_BET_, Brunauer–Emmett–Teller (BET) specific surface area; V_t_, total pore volume; D_BJH_, Barrett–Joyner–Halenda (BJH) pore diameter.

**Table 2:** Results of loading content (LC) and encapsulation efficiency (EE) of AZOX and Dini into NH_2_-Al-MIL-101.

Entry	Carrier Material	Pesticide	LC (%)	EE (%)
Entry 1	NH_2_-Al-MIL-101	AZOX	7.28 ± 0.34	9.17 ± 0.42
Entry 2	NH_2_-Al-MIL-101	Dini	29.79 ± 0.67	42.99 ± 0.97
Entry 3	NH_2_-Al-MIL-101	AZOX	6.71 ± 0.46	7.19 ± 0.53
		Dini	29.72 ± 0.29	42.24 ± 0.59

Dichloromethane was used as the solvent for loading pesticides. Values are mean ± SD of three replicates.

**Table 3:** Correlation coefficient, rate constant, and half-life obtained by fitting the release data of AZOX and Dini from AZOX@Dini@NH_2_-Al-MIL-101 at different pH values of 3.0, 7.1, and 10.1 using five different kinetic mathematical models.

pH	Fitting Model	AZOX		Dini	
		Kinetic Equation	R^2^	Kinetic Equation	R^2^
3.0	Zero-order	y = 50.86 + 1.28t	0.5390	y = 11.95 + 0.60t	0.9798
	First-order	y = 87.53 × (1−e^(−0.31t)^)	0.9814	y = 110.11 × (1 − e^(^^−^^0^^.01t)^)	0.9854
	Korsmeyer–Peppas	y = 39.61 × (t^0.25^)	0.8485	y = 3.45 × (t^0.66^)	0.9946
	Higuchi	y = 11.18 × (t^1/2^) + 31.54	0.7408	y = 8.02 × (t^1/2^) −8.80	0.9812
7.1	Zero-order	y = 68.91 + 0.93t	0.3445	y = 25.33 + 0.62t	0.7980
	First-order	y = 95.62 × (1−e^(−0.49t)^)	0.9846	y = 94.27 × (1 − e^(^^−^^0^^.03t^)	0.9961
	Korsmeyer–Peppas	y = 56.52 × (t^0.17^)	0.9677	y = 10.37 × (t^0.46^)	0.9447
	Higuchi	y = 8.65 × (t^1/2^) + 52.92	0.7384	y = 895 × (t^1/2^) −0.40	0.9414
10.1	Zero-order	y = 38.27 + 0.66t	0.3360	y = 14.13 + 0.70t	0.9312
	First-order	y = 57.76 × (1 − e^(^^−^^0^^.40t)^)	0.9922	y = 112.41 × (1 − e^(^^−^^0^^.01t)^)	0.9928
	Korsmeyer–Peppas	y = 30.37 × (t^0.20^)	0.7027	y = 4.63 × (t^0.63^)	0.9793
	Higuchi	y = 6.16 × (t^1/2^) + 26.89	0.5453	y = 9.48 × (t^1/2^) −11.26	0.9810

**Table 4:** Calculated EC_50_ of pure AZOX, pure Dini, physical mixture of AZOX and Dini (AD) and AZOX@Dini@NH_2_-Al-MIL-101 (PDS) against rice sheath blight (*R. solani*) at day 2 incubated at 25 ± 2 °C.

Fungicides	Toxicity Regression Equation	Correlation Coefficient	EC_50_(ob) (mg/mL)	EC_50_(th) (mg/mL)	Synergistic Ration
AZOX	y = −0.04 + 0.49x	0.904	1.251	−	−
Dini	y = 1.53 + 1.56x	0.984	0.099	−	−
AD	y = 0.89 + 0.85x	0.998	0.087	0.1187	1.36
PDS	y = 1.17 + 0.98x	0.994	0.065	0.1192	1.83

## Data Availability

The data presented in this study are available in this article.
